# First and second year medical students identify and self-stereotype more as doctors than as students: a questionnaire study

**DOI:** 10.1186/s12909-017-1049-2

**Published:** 2017-11-13

**Authors:** Bryan Burford, Harriet E. S. Rosenthal-Stott

**Affiliations:** 1 School of Medical Education, Newcastle University, Newcastle upon Tyne, NE2 4HH UK; 2 Department of Psychology, Durham University, Durham, UK

**Keywords:** Professional identity, Stereotypes, Transitions, Professional development, Recruitment, Widening participation

## Abstract

**Background:**

The emergence of medical students’ professional identity is important. This paper considers this in a snapshot of the early years of undergraduate medical education. From the perspective of social identity theory, it also considers self-stereotyping, the extent to which individuals associate with attributes identified as typical of groups.

**Method:**

Paper questionnaires were completed by first and second year medical students following teaching sessions at the beginning (October) and end (April) of the academic year. Questionnaires consisted of scales measuring the strength and importance of identity and self-stereotyping, referent to ‘doctors’ and ‘students’. Linear mixed effects regression considered longitudinal and cross-sectional effects of progress through the course, and differences in responses to ‘doctor’ and ‘student’ measures.

**Results:**

In October, responses were received from 99% (*n* = 102) and 75% (*n* = 58) of first and second year cohorts respectively, and in April from 81% (*n* = 83) and 73% (*n* = 56). Response rates were over 95% of those present.

Linear mixed effects regression found that all ‘doctor’-referent measures were higher than ‘student’ measures.

Strength of identity and self-stereotyping decreased between beginning and end of the year (across both groups). Men indicated lower importance of identity than women, also across both groups. There were no differences between year groups.

Self-stereotyping was predicted more by importance of identification with a group than by strength of identification.

**Conclusions:**

Findings reinforce observations that medical students identify strongly as doctors from early in their studies, and that this identification is greater than as students.

Decreases over time are surprising, but may be explained by changing group salience towards the end of the academic year. The lack of a gender effect on strength of identification contrasts with the literature, but may reflect students’ lack of ‘performance’ of professional identity, while the effect on importance is speculated to be linked to social identity complexity.

Identification with professional group may have implications for how medical schools treat students. The findings on self-stereotyping have relevance to recruitment if applicant populations are limited to those already internalising a stereotype. There may be consequences for the wellbeing of those who feel they cannot fulfil stereotypes when in training.

**Electronic supplementary material:**

The online version of this article (10.1186/s12909-017-1049-2) contains supplementary material, which is available to authorized users.

## Background

The subject of medical students’ and doctors’ professional identity is increasingly of interest in medical education research. It is now recognised that a developing sense of ‘being a doctor’ has important implications for professional and personal development, influencing how professionals practice and learn [1]. It has recently been posited as the essence of professionalism – that becoming a doctor is the key to professional practice [2]. Identity also plays a role in medical students’ well-being [3], with a strong identity potentially having a protective effect against stress [4].

In this paper, we consider the professional identity of pre-clinical medical students, and in particular how this can be considered in relation to the stereotypes held by students. We adopt the explanatory framework and vocabulary of the social identity approach (comprising social identity theory [5] and self-categorisation theory [6]). While not the only possible theoretical approach, it is gaining popularity as a framework for examining professional identity in a medical education setting [7–9]. Fundamental to this approach is that while individuals may belong to many different groups (e.g., doctor, student, female), the dominance or *salience* of a particular group – that is the extent to which an individual subjectively identifies with it and to which it influences their perceptions and behaviour – is variable. Medical students are potentially members of two distinct groups related to their work: their professional group of ‘doctor’, and their ‘student’ group, and we consider both of these in this paper.

Studies have shown that medical students and other health care professional students identify with their professional group early in undergraduate training [10, 11]. It has been suggested that professional and student identities are in tension, with medical education aiming to move the student towards the professional ‘doctor’ identity, while at the same time focusing on their ‘student’ identity [12]. Others have suggested that medical students are required to think either ‘like a student’ or ‘like a doctor’, depending on context [13] and that there is a slow transition from student to doctor, with a developing feeling of inclusion as a member of the professional group, concurrent with increasing exclusion from the student group [14]. Gender differences have been noted in the development of professional identity in a number of professions including medicine, with women observed to report stronger identification than men [10, 11]. Theoretical explanation for this is limited, but it has been suggested it may be related to the way in which identity is performed in interprofessional interactions [10].

This paper adds to this literature, by examining how medical students’ identification as a doctor compares to identification as a student at different points in the first two years of study. It also introduces a theoretically important element: stereotyping. Stereotyping is an important concept in understanding how groups are socially constructed and is used in many ways [15]. Self-categorization theory suggests that when individuals identify with a group they will perceive themselves as possessing attributes associated with the group, referred to as self-stereotyping [16]. A stereotype in this sense is not necessarily negative, but rather a set of attributes associated with a group. Beginning to understand if, and how, medical students associate themselves with stereotypes of doctors may further illuminate not just the development of professional identity but also of professional attitudes and professional behaviours – in short, professionalism.

In this study, we aimed simply to establish how medical students’ identification and self-stereotyping with the groups ‘student’ and ‘doctor’ varied with progression towards being a qualified doctor both longitudinally within the academic year, and cross-sectionally between years. Student and doctor are among many identities potentially available to individuals, but are those which may be specifically salient to all medical students in the context of medical school.

We also considered how these measures varied with students’ career plans or aspirations at this early stage, with the hypothesis that those with more developed ideas on eventual specialty may have more considered opinions on how well a doctor identity ‘fits’, meaning their identity measures will differ from those who do not have a clear preference.

## Method

This was a questionnaire study with data collected from two student cohorts at two time points. Participants were first and second year medical students at a UK medical school which delivered the first 2 years of a five-year medical degree. Both years are pre-clinical, but include hospital and GP visits as part of an integrated curriculum. There were 103 students in year 1 at the time of the study, and 78 in year 2.

### Dependent variables

The questionnaire completed by participants is available as Additional file 1 attached to this paper. Details of measures are given below.

### Group identification

Group identification was measured on two validated scales. The first was a modification of a four-item subscale which examined the importance of group membership [17] and the second was a 10-item scale widely used in the social identity literature, particularly in organisational contexts [18]. This asks respondents to rate a wider range of affective elements of association with the group, but is treated as a single scale effectively indicating ‘strength’ of identification. It has previously been used with doctors and nurses [19] and with medical students [10, 11].

Two versions of the scales were completed – one referring to ‘doctors’ as the group membership in question, and the other to ‘students’. For both measures, responses were made on a 7-point scale, with anchors ‘strongly disagree’ and ‘strongly agree’.

### Stereotyping

Assessing the extent of self-stereotyping requires a method of ascertaining stereotypical attributes. It is important to note that a stereotype is not a fixed entity, rather it is a set of attributes associated with a group, which may vary idiosyncratically, or with cultural context. Here we adapted a method from the literature for identifying and measuring stereotypical attributes of a group and measuring an individual’s self-stereotyping against those attributes [20].

A pre-test procedure used an online form to elicit attributes associated with the groups ‘doctors’ and ‘students’ from a sample of 56 students of different disciplines across university faculties. While this was a convenience sample, the intention was not to provide a definitive set of attributes but rather to source attributes based on the perceptions of a group drawn from the wider population including non-medics. Thirteen of the respondents were medical students, and there is a chance that some of these, if they were in first year at that point, would have been in second year during main data collection. However, given the questionnaire data collection was independent and several months later, any confounding is minimal. Only attributes which were used to describe just one or other group were retained in the main study questionnaire.

The ‘doctor’ attributes generated by the participants in this exercise were all ostensibly positive. In order to provide some balance in the questionnaire, an additional seven negative attributes associated with doctors were selected from the literature [21, 22] and included in the questionnaire. Five generic positive and five negative items from earlier work were also included [23]. This gave a total set of 47 descriptors which were randomly sorted for presentation in the questionnaire.

The questionnaire contained three sets of questions to derive the measure of self-stereotyping. Each attribute was rated by participants firstly in terms of how true it was of themselves (on a 7-point scale, ranging from 1 – ‘Not at all true of me’ – to 7 – ‘Very true of me’), secondly in terms of how representative it was of doctors (‘please indicate the percentage of doctors who you think possess each characteristic’ with responses on an 11-point scale labelled 0% to 100%) and finally how representative of students (using the same scale as for doctors).

### Demographics and pseudonymising code

Finally, respondents were asked basic demographic information (age, gender, nationality and ethnicity) in order to provide a demographic profile of respondents, and what specialty career path they intend to pursue as a doctor. Specialty was operationalised as seven broad specialty categories which junior medical students were likely to be familiar with, but with options for ‘Other’ and ‘I haven’t decided yet’.

In order to potentially match responses at the two time-points, respondents were also asked to generate a pseudonymising code, consisting of the initial of their first name, the day of their birthday (e.g., 22), and the first two letters of the town/city where they were born.

### Procedure

Participants completed questionnaires twice – once at the beginning of the academic year in the third and fourth week of term in October, and once at the end of the teaching year in April. At both time points, the questionnaire was administered in lecture theatres following a teaching session, at which the majority of each year group were present, although sessions were not compulsory and registers were not taken. The study was introduced at the end of teaching and the questionnaire distributed by BB, with a verbal briefing stressing that completion was optional. Students then completed and returned the questionnaire, taking 10–20 min to do so.

### Analysis

Data were analysed using mixed effects regression modelling with the ‘lmerTest’ package [24] in the R statistical programming environment [25]. Mixed effects modelling allows all data to be used, as it does not require listwise deletion of cases where just one item of data may be missing. A helpful, non-technical, primer for such analysis can be found online [26]. lmerTest provides an estimated *p*-value for regression coefficients. The reporting of *p*-values is contentious in mixed effects modelling, but aids interpretation.

Analyses on each of the variables included a random intercept for respondent, and random slope by respondent across Target Group – this means that the model allows the intercept and slope of individual respondents to vary with regard to the difference between ‘doctor’ and ‘student’ responses, effectively controlling for individual variability.

Criterion-based model selection used the anova() function in R to compare the fit of models [27]. Initial models included gender, target group, time of year, year of course and whether a student expressed a preference for any specialty as fixed factors. The contributions of two-way interaction terms to these models were considered to identify if effects were consistent between men and women, between first and second years and between October and April data collection. No interaction terms significantly improved fit and so were not considered further. The contribution of each individual term was then considered. Specialty preference did not significantly contribute to any models. Time, gender and year contributed differently to models for each variable, and effects were retained only when significant. The R code for these analyses is available from the authors.

Analysis of the self-stereotyping scales followed Biernat et al. [20], with the self-ratings recoded into a 0–100 scale (using the equation [score - 1] × 16.6667). A mean was calculated for each subset of attributes (ie, ‘doctor’, ‘student’, ‘negative doctor’, ‘generic positive’, ‘generic negative’), and two overall indices of self-stereotyping calculated for each respondent by correlating self-ratings against each attribute with the doctor and student group ratings.

## Results

### Participants

At the beginning of the year in October, 102 first years (99% of cohort) and 58 second years (75% of cohort) completed the questionnaire. In April, completed questionnaires were returned by 83 first years (81%) and 56 second years (73%). These figures represented estimated response rates of >95% of the students present at each session.

Demographics, where responses were given, were as follows: in October, 50 first year respondents (51% of those who gave an answer) indicated they were male and 48 female (49%), and in April 42 male (52%) and 38 female (48%). For second years, the figures were 25 male (43%), 33 female (57%) in October and 27 male (51%), 26 female (49%) in April. In October, the age of first years ranged from 17 to 42 (median = 19, IQR = 3), and of second years 19 to 36 (median 20, IQR = 5). In October, 7 first years (7%) and 9 second years (15%) were over 25 years old.

Overall, the majority of respondents (256, or 90% of those who gave an answer) identified as British (including dual nationalities and ‘British Asian’) and as White (183, 66% of responses).

The majority of responses were ‘undecided’ about speciality, and there was little change between the beginning and end of the year: 64 first years and 31 second years were undecided in October (63% and 53% respectively), and 54 first years and 30 second years in April (65% and 54%). Most of those who gave a preference indicated the ‘medicine’ or ‘surgery’ categories (first years: 13% and 14% respectively in October, 7% and 16% in April; second years: 16% and 10% in October, 18% and 7% in April).

In October, 144 participants (90 first year, 54 second year) provided a complete identifier allowing questionnaires to be linked, but in April just 123 did so (76 first year, 47 second year) and of these just 91 (60 first year, 31 second year) matched identifiers from October – suggesting the code was not as robust as hoped. Twenty-four responses (12 in October, 12 in April) did not include a pseudonymising code at all, and were allocated unique identifiers for inclusion in analysis.

### Effects on identity measures

Coefficients from the regression models for both identity scales are given in Table 1, with their 95% confidence intervals. Where coefficients are not given for a factor, it means the effect was not retained following the model selection procedure described in the method. Coefficients indicate the change in the outcome variable associated with the change in level of each factor (effectively the mean difference between levels within the model, not the sample means). For both measures, there is a significant difference between scores relating to student and doctor identities, with those for doctor identity being higher. Additionally, men score lower on the importance scale than women, while strength scores are lower at the end of the year than at the beginning (sample means are in Table 2).

**Table 1:** Regression coefficients for mixed effects regression analyses on identity measures

Variable	Coefficient ^a^	95% CI around coefficient	*p*-value
Importance			
Target group (doctor compared to student)	0.52	0.34 to 0.70	*p* < 0.0001
Gender (female compared to male)	0.47	0.20 to 0.75	*p* < 0.001
Strength			
Target group (doctor compared to student)	0.22	0.09 to 0.35	*p* < 0.001
Time (April compared to October)	−0.34	−0.43 to −0.25	*p* < 0.0001

^a^Coefficients use the units of the outcome measure, in this case the 7-point scale

**Table 2:** Sample means and standard deviations for identity measures

	Mean (sd) for Importance scale	Mean (sd) for Strength scale
Target Group	Doctor: 4.67 (1.18); Student: 4.18 (1.31) ^***^	Doctor: 5.42 (0.68); Student: 5.21 (0.98) ^***^
Gender	Female: 4.64 (1.18); Male: 4.22 (1.32) ^***^	Female: 5.39 (0.77); Male: 5.24 (0.92)
Year	Year 1: 4.51 (1.17); Year 2: 4.29 (1.41)	Year 1: 5.37 (0.79); Year 2: 5.22 (0.94)
Time	Time 1: 4.44 (1.30); Time 2: 4.41 (1.24)	Time 1: 5.45 (0.77); Time 2: 5.15 (0.91) ^***^

^***^p < = 0.001

### Stereotype measures

#### Construct validity

Before examining self-stereotyping, we confirmed that each set of attributes included in the questionnaire were associated with the intended group – that is, on the items asking what percentage of each group possesses each attribute, the ‘doctor’ attributes were associated more with doctors as a group and the ‘student’ attributes more with students. T-tests were carried out on the scores of each of the attributes at Time 1, comparing the mean association by respondents of each with ‘doctor’ and ‘student’ groups. Because of the large number of simultaneous tests, a Bonferroni correction was applied to interpretation of *p*-values such that 5% confidence is indicated by *p* < 0.001. Table 3 summarises the percentage ratings for all attributes at the first time point.

**Table 3:** Association of each attribute with doctors and students: mean (standard deviation) percentage

Attribute	Doctor	Student
Student attributes ^a^		
Lazy	22.4 (15.4)	50.4 (22.7)*
Fun	51.1 (16.6)	70.4 (11.7)*
Happy	57.5 (17.2)	66.8 (14.9)*
Loud	42.5 (18.9)	62.4 (15.1)*
Poor	10.6 (11.8)	40.4 (25.0)*
Young	34.8 (15.4)	79.1 (13.1)*
Drinker	50.7 (23.2)	74.7 (17.5)*
Outgoing	59.8 (16.8)	70.9 (12.8)*
Relaxed	51.4 (21.3)	53.4 (19.6)
Naive	16.3 (14.5)	47.1 (22.1)*
Sporty	50.5 (16.4)	61.3 (15.1)*
Independent	74.5 (19.6)	57.6 (20.2)*
Carefree	24.8 (17.5)	52.8 (22.1)*
Doctor attributes ^a^		
Approachable	69.5 (17.3)	58.9 (16.9)*
Calm	70.1 (18.4)	43.6 (17.8)*
Committed	79.8 (14.1)	60.5 (19.2)*
Compassionate	76.7 (14.0)	57.6 (16.8)*
Considerate	70.8 (15.0)	59.3 (16.2)*
Empathetic	76.2 (15.4)	55.2 (16.0)*
Honest	78.2 (15.0)	58.1 (17.7)*
Kind	71.9 (14.7)	62.5 (15.9)*
Knowledgeable	87.1 (11.3)	56.3 (17.9)*
Logical	81.4 (12.4)	57.8 (18.0)*
Patient	70.6 (17.2)	49.2 (17.5)*
Professional	86.6 (12.5)	49.4 (20.0)*
Reliable	79.2 (1.03)	56.1 (16.5)*
Responsible	83.4 (11.8)	54.3 (17.9)*
Trustworthy	80.1 (13.6)	57.8 (17.7)*
Understanding	77.8 (13.3)	62.1 (15.0)*
Wealthy	75.2 (16.1)	45.7 (20.3)*
Negative doctor attributes ^b^		
Domineering	39.9 (23.4)	33.7 (20.4)
Aggressive	14.4 (14.9)	22.6 (17.7)*
Dithering	22.2 (16.8)	36.5 (20.8)*
Confused thinker	16.9 (14.7)	35.8 (20.0)*
Emotionally unstable	21.1 (16.2)	30.5 (20.1)*
Arrogant	48.9 (23.2)	45.1 (21.0)
Detached	35.9 (23.1)	27.8 (16.9)
Generic positive attributes ^c^		
Warm	62.4 (16.4)	59.8 (15.6)
Good-natured	70.9 (16.8)	66.2 (14.9)
Truthful	77.1 (15.6)	57.6 (17.5)*
Loyal	69.1 (16.1)	55.1 (16.9)*
Honourable	78.0 (13.8)	52.4 (17.7)*
Generic negative attributes ^c^		
Liar	15.7 (14.4)	26.7 (17.6)*
Hostile	19.4 (16.4)	25.4 (16.6)
Cruel	8.1 (11.4)	13.3 (14.3)
Spiteful	17.0 (15.5)	25.0 (19.2)*
Abusive	8.2 (10.8)	16.2 (16.7)*

^a^Derived from pre-test procedure

^b^Derived from historical literature

^c^Derived from Rosenthal et al. 2006

*p < 0.001 for difference between Doctor and Student ratings

All 17 pre-test generated ‘doctor’ attributes were associated more with doctors (p < 0.001), and all but one of the 13 ‘student’ attributes were associated more with students (p < 0.001). The validity of these attributes as representing stereotypes for the participant sample is therefore demonstrated. The other groups – which had not been generated directly from data – were less valid. Of the ‘negative doctor’ set, derived from historical literature, only one was associated more with doctors than students. Four of the five generic negative attributes were associated more with students, and four of the five generic positive attributes with doctors. This is in itself interesting and will be returned to in the discussion.

### Self-stereotyping

To calculate self-stereotyping against these attributes, aggregated self-ratings for the validated sets of ‘doctor’ (17 items) and ‘student’ (12 items) attributes were compared. Means were calculated for each of the sets, and t-tests carried out. The mean for self-rating against the ‘doctor’ attributes was significantly higher than that for the ‘student’ attributes: doctor mean = 73.85 (sd = 8.19), student mean = 52.09 (sd = 9.63; t(135) = 21.67, *p* < 0.0001).

However, this aggregation may mask individual variations (some individuals associated some ‘doctor’ attributes more with students, and vice versa). Further analysis therefore considered individual indices of self-stereotyping against ‘doctor’ or ‘student’ attributes (the correlation between individuals’ self-ratings and their ratings of all attributes as representative of doctors and students). As with the identity measures, mixed effects regression could then be carried out on these measures. Table 3 contains the coefficients from this analysis (Table 4).

**Table 4:** Coefficients for mixed effects regression of self-stereotyping index

Variable	Coefficient	95% CI around coefficient	p-value
Target group (doctor compared to student)	0.22	0.18 to 0.26	*p* < 0.0001
Time (April compared to October)	−0.05	−0.07 to −0.02	*p* < 0.001

*Coefficients use the units of the outcome measure, in this case the self-stereotyping index on a scale −1 to 1

This shows that as for strength of identity, self-stereotyping against ‘doctor’ ratings was significantly higher than against ‘student’ ratings, and decreased between the beginning and end of the academic year. Sample means are given in Table 5.

**Table 5:** Sample descriptive statistics for self-stereotyping index

	Mean (sd) for Self-stereotyping index
Target Group	Doctor: 0.64 (0.20), Student: 0.42 (0.34) ***
Gender	Female: 0.52 (0.29), Male: 0.53 (0.31)
Year	Year 1: 0.55 (0.30), Year 2: 0.50 (0.30)
Time	Time 1: 0.56 (0.28), Time 2: 0.50 (0.33) ***

***p < = 0.001

### Self-stereotyping and identity

Self-stereotyping is suggested to be linked conceptually, and cognitively, with identity. To consider the relationship between the three measures, a further regression was carried out, with the two identity measures as predictors of self-stereotyping. Target Group was also included as a fixed effect, and respondent with random intercept and random slope across Target.

Strength, but not importance, was a significant predictor of self-stereotyping index, with a coefficient of 0.09 (*p* < 0.001, 95% CI around coefficient 0.07 to 0.12), compared to <0.01 (95% CI -0.02 to 0.01) for Importance.

## Discussion

We set out to examine the professional identity of medical students in their first two years of study, referent to two groups that may be salient to them: students and doctors. We also considered the extent to which they self-stereotype, or associate themselves with stereotypical attributes of doctors or students.

We established that medical students identified more with their doctor identity than with their student identity, supporting findings in the literature. The doctor identity is also more important to them. This is important as it suggests that medical students view themselves as doctors, above the alternative, and objectively more valid, identity of ‘student’.

We have considered two identity scales which demonstrate slightly different effects. The importance of both doctor and student identities varies with gender, while their strength does not. An effect of gender on the strength scale had been anticipated due to earlier findings in the literature [10, 11], but this effect was not observed. The effect had been suggested as being a consequence of the performance of identity in interprofessional interactions being different for men and women [10], and it may be that as there had been little opportunity for first or second year medical students to perform the ‘doctor’ role, the stimulus for divergence was not present. The student identity meanwhile is less related to interpersonal interactions, and so such an effect may not be expected.

The difference in the importance scale – with women rating both groups as more important – may be a measurement artefact, but may imply that men have other, more subjectively important identities that were not measured here. We can speculate that the concept of social identity complexity – essentially the number of available identities and how they interact [28] – may be a factor. If the male students had greater social identity complexity, they may judge the relative importance of doctor and student groups to be lower. Although gender effects on social identity complexity do not appear to have been widely considered in the literature, this may be worth further study. The similar concept of self-complexity has also been linked to wellbeing in medical students [3].

Strength of identification with both groups reduces through the course of the academic year, against expectations. That both year groups were close to end of year examinations when the second questionnaire was completed may be behind this, if uncertainty and anxiety about the outcome of those exams challenged their identities. It may also simply be that towards the end of the year, group membership associated with medical school is less salient as students look beyond exams to the summer vacation.

That there is no difference between year groups is surprising, given that second years are substantially closer to the milestone of qualification as a doctor, and may be expected to have consolidated a student identity in having an additional year. This suggests that medical students may arrive at medical school with doctor and student identities already established, something which may reflect ‘anticipatory categorisation’ [29]. It is also interesting and pertinent that the importance of both groups remains static.

This may have implications for well-being, as previous research has established that identity and well-being are linked, with a strong identity providing a buffer of resilience [3, 4]. Monitoring students’ levels of identification early during medical school may indicate their future well-being and it may be that techniques to raise identification (e.g., through priming [30, 31]), could promote future well-being. Points at which group membership is challenged – for example difficulties with assessment – may also be viewed through the lens of social identity in order to facilitate support. However, unintended adverse consequences of strong professional identity should not be ruled out, such as overconfidence or lowered attention to learning.

Medical students also describe themselves more in line with attributes stereotypically associated with doctors than students. This self-stereotyping reinforces the relevance of self-categorisation theory [6], which suggests that when people identify with a group, they will describe themselves in line with the stereotype of the group. The literature suggests that newcomers to a group are more likely to self-anchor – that is project their personal attributes onto the group – than self-stereotype [29, 32]. However, as doctors constitute a well-known and well-established group, self-stereotyping seems to be the more likely explanation here. Self-stereotyping can be seen as effectively a mechanism by which to improve ‘normative fit’, the perceived match between a group and an individual [33].

It is worth noting that self-stereotyping is more associated with lower status groups, and acts as a ‘protection’ of self-perception [34]. While ‘doctors’ are a high status group, our findings may also reflect medical students’ lower status in the medical hierarchy, compared to qualified doctors, and an implicit boost to esteem through assimilating the desired group identity. Repeating the study with qualified doctors could explore this point, but it seems there is a possible theoretical explanation for our findings.

The effects observed on self-stereotyping mirrored those on strength of identity, which was found to be a significant predictor of self-stereotyping, while Importance of identity was not. This suggests a more refined view of the concept of identity, and its relationship with cognitive processes, may be appropriate. In future work we will look to a possible three factor model of identity [35] which differentiates between centrality (cognitive accessibility of the identity; how often the identity is thought about – analogous to importance); ingroup affect (feelings associated with group membership); and ingroup ties (sense of belonging with other group members – analogous to strength). Further examination of the cognitive processes behind stereotyping in medicine may be useful here.

### Content of stereotypes and professionalism

Finally, notwithstanding the limited scope of the pre-test, it is worth commenting on the fact that the attributes identified as typical of doctors by a sample of students were all positive. In addition, a set of negative attributes which had been associated with doctors in the literature, and a set of ‘generic’ negative attributes, were more associated with students. This suggests that despite challenges to the public image of medicine and healthcare, the underlying perception of doctors is still positive.

Medical students also viewed doctors positively, and perceived themselves as having those positive attributes. While these perceptions may not always translate into behaviour, this is an encouraging finding in relation to students’ professionalism. Professional identity has recently been linked to the concept of professionalism [2], and it may be that the internalisation of positive stereotypes through self-stereotyping is a mechanism by which professionalism is instantiated in identity.

### Limitations

Our study does have some limitations. Firstly, only a single UK medical school was involved. While studies of identity across medical schools have not been carried out, we know that on some key variables, such as preparedness, there are differences [36]. We have no theoretical reason to expect the patterns observed to vary, but nonetheless, further work comparing medical schools will be helpful. Further, this was an undergraduate medical programme, and while some of the older students may have been graduates, programmes and systems with entirely graduate entry may show different results. Individual differences in familiarity with medicine, such as through family connections, are also not considered.

Secondly, we considered only the first two years of undergraduate medical education. While this provides an important initial view of identity development, we cannot anticipate what happens later on in the degree programme. In particular, in their third year, medical students enter clinical placements, and in this particular programme spend their entire third and fifth years based in hospitals. This change in environment may influence their identification with ‘doctor’ group membership. However, the direction of this influence is uncertain. Working in the clinical environment may increase opportunities to perform the doctor identity, meaning medical students feel more like doctors, and thus increasing identification. However, it may provide more contrasts with other groups (e.g., qualified doctors, nurses, other healthcare professionals, and patients) which emphasise the *student* identity, and so provide challenges which may undermine professional identity.

The questionnaire’s phrasing of questions with reference to ‘students’ rather than ‘medical students’ may omit an important category. Anecdotally, medical students feel distinct from other students, and their experiences, and vocational focus, differ from many other undergraduates. ‘Student’ also has associations with pre-university education which may dilute its meaning. Questions phrased with reference to ‘medical students’ may have elicited different responses.

Finally, the stereotype measure was generated from disparate sources. The pre-test data was generated from a sample of students, albeit from a number of subject areas. A wider sample of the general public, containing different ages and levels of education, may have uncovered a more varied set of attributes. However, the content of the set of attributes was not a primary focus of the study, and the index of self-stereotyping is not affected by individual views of individual attributes.

## Conclusion

We have confirmed that medical students identify as doctors from as early as their first few weeks at university. We have also established that this identity is more important to their sense of self than their student identity, and that they self-stereotype as doctors – meaning they feel attributes associated with doctors apply to them – more than they self-stereotype as students. In essence, medical students consider themselves to be doctors and describe themselves as typical doctors, rather than as typical members of the undergraduate student body.

The implications of this for undergraduate medical education need some consideration. On one hand, if medical students feel like doctors, they should perhaps be treated as such – as *trainee doctors* rather than medical *students* – to encourage their professional development. On the other hand, there are potential risks of overconfidence from a professional identity that is over-developed in relation to an individual’s level of training and statutory position; students should not be encouraged to over-reach their competence. There is also a risk that identifying less as a student may reflect perceptions of learning as being distinct from being a doctor, despite medical practice containing the need to be a lifelong learner.

The findings on self-stereotyping may also have relevance to recruitment and widening participation in medicine. If those who enter medicine begin by feeling they are similar to a stereotype, it may limit the population of applicants to those who feel they are capable of entering medicine. Those who feel they cannot fulfil stereotypical characteristics once in medical education may also feel challenged, with effects on wellbeing. Examining the longer-term perceptions of identity and self-stereotyping among medical students as they progress through medical school and into practice, and of those who fail or drop out, would clarify this.

## Additional file

Additional file 1: The full questionnaire is available in the supplementary file prof_id_stereotyping_q.docx. (DOCX 68 kb)
