# Supplementary material for: First and second year medical students identify and self-stereotype more as doctors than as students: a questionnaire study
Source: BMC Med Educ. 2017 Nov 13;17:209. doi: 10.1186/s12909-017-1049-2 (PMC5683566; doi:10.1186/s12909-017-1049-2)
Supplement: Additional file 1: — The full questionnaire is available in the supplementary file prof_id_stereotyping_q.docx. (DOCX 68 kb) [file 12909_2017_1049_MOESM1_ESM.docx]

[Full questionnaire as used by Burford & Rosenthal Stott 2017. Details of sources and derivation of scales are provided in full paper]

**CONSENT FORM**

**Participant Information**

**We are interested in how you view yourself and how you view other groups of people.**

**In this questionnaire you will be asked some questions about how you perceive yourself and others.**

**All of the information you give will remain confidential and anonymous.**

**All questions are optional. You have the option to omit any question you do not wish to answer**.

*Please cross out*

*as necessary*

Have you read the Participant Information above? YES / NO

Do you consent to participate in the study? YES / NO

Do you understand that you are free to withdraw from the study:

* at any time and

* without having to give a reason for withdrawing and

* without any adverse result of any kind? YES / NO

**Signed** .............................................………................ **Date** ...........................................

(NAME IN BLOCK LETTERS) ......................................................………........................

**PLEASE REMOVE THIS SHEET FROM THE REST OF THE QUESTIONNAIRE**

**IN ORDER TO RETAIN ANONYMITY**

**The study is being conducted by:**

**[research details here]**

**If you have any queries contact [lead researcher email]**

1. Please **circle** the number to indicate how much you think each of the following traits is true of **YOU**.

|  | **Not at all Very true**  **true of me of me** |
| --- | --- |
| 1. Fun | 1 2 3 4 5 6 7 |
| 1. Loud | 1 2 3 4 5 6 7 |
| 1. Calm | 1 2 3 4 5 6 7 |
| 1. Happy | 1 2 3 4 5 6 7 |
| 1. Committed | 1 2 3 4 5 6 7 |
| 1. Honourable | 1 2 3 4 5 6 7 |
| 1. Drinker | 1 2 3 4 5 6 7 |
| 1. Wealthy | 1 2 3 4 5 6 7 |
| 1. Loyal | 1 2 3 4 5 6 7 |
| 1. Professional | 1 2 3 4 5 6 7 |
| 1. Relaxed | 1 2 3 4 5 6 7 |
| 1. Hostile | 1 2 3 4 5 6 7 |
| 1. Truthful | 1 2 3 4 5 6 7 |
| 1. Honest | 1 2 3 4 5 6 7 |
| 1. Arrogant | 1 2 3 4 5 6 7 |
| 1. Approachable | 1 2 3 4 5 6 7 |
| 1. Responsible | 1 2 3 4 5 6 7 |
| 1. Logical | 1 2 3 4 5 6 7 |
| 1. Spiteful | 1 2 3 4 5 6 7 |
| 1. Detached | 1 2 3 4 5 6 7 |
| 1. Abusive | 1 2 3 4 5 6 7 |
| 1. Independent | 1 2 3 4 5 6 7 |
| 1. Cruel | 1 2 3 4 5 6 7 |
| 1. Poor | 1 2 3 4 5 6 7 |
| 1. Empathetic | 1 2 3 4 5 6 7 |
| 1. Compassionate | 1 2 3 4 5 6 7 |
| 1. Patient | 1 2 3 4 5 6 7 |
| 1. Outgoing | 1 2 3 4 5 6 7 |
| 1. Young | 1 2 3 4 5 6 7 |
| 1. Considerate | 1 2 3 4 5 6 7 |
| 1. Kind | 1 2 3 4 5 6 7 |
| 1. Aggressive | 1 2 3 4 5 6 7 |
| 1. Trustworthy | 1 2 3 4 5 6 7 |
| 1. Carefree | 1 2 3 4 5 6 7 |
| 1. Reliable | 1 2 3 4 5 6 7 |
| 1. Domineering | 1 2 3 4 5 6 7 |
| 1. Naïve | 1 2 3 4 5 6 7 |
| 1. Knowledgeable | 1 2 3 4 5 6 7 |
| 1. Dithering | 1 2 3 4 5 6 7 |
| 1. Confused thinker | 1 2 3 4 5 6 7 |
| 1. Sporty | 1 2 3 4 5 6 7 |
| 1. Warm | 1 2 3 4 5 6 7 |
| 1. Emotionally unstable | 1 2 3 4 5 6 7 |
| 1. Good-natured | 1 2 3 4 5 6 7 |
| 1. Liar | 1 2 3 4 5 6 7 |
| 1. Understanding | 1 2 3 4 5 6 7 |
| 1. Lazy | 1 2 3 4 5 6 7 |
|  | **Not at all Very true**  **true of me of me** |

1. Please **tick** to indicate the percentage of **DOCTORS** who you think possess each characteristic

|  | 0% | 10% | 20% | 30% | 40% | 50% | 60% | 70% | 80% | 90% | 100% |
| --- | --- | --- | --- | --- | --- | --- | --- | --- | --- | --- | --- |
| 1. Fun | ☐ | ☐ | ☐ | ☐ | ☐ | ☐ | ☐ | ☐ | ☐ | ☐ | ☐ |
| 1. Loud | ☐ | ☐ | ☐ | ☐ | ☐ | ☐ | ☐ | ☐ | ☐ | ☐ | ☐ |
| 1. Calm | ☐ | ☐ | ☐ | ☐ | ☐ | ☐ | ☐ | ☐ | ☐ | ☐ | ☐ |
| 1. Happy | ☐ | ☐ | ☐ | ☐ | ☐ | ☐ | ☐ | ☐ | ☐ | ☐ | ☐ |
| 1. Committed | ☐ | ☐ | ☐ | ☐ | ☐ | ☐ | ☐ | ☐ | ☐ | ☐ | ☐ |
| 1. Honourable | ☐ | ☐ | ☐ | ☐ | ☐ | ☐ | ☐ | ☐ | ☐ | ☐ | ☐ |
| 1. Drinker | ☐ | ☐ | ☐ | ☐ | ☐ | ☐ | ☐ | ☐ | ☐ | ☐ | ☐ |
| 1. Wealthy | ☐ | ☐ | ☐ | ☐ | ☐ | ☐ | ☐ | ☐ | ☐ | ☐ | ☐ |
| 1. Loyal | ☐ | ☐ | ☐ | ☐ | ☐ | ☐ | ☐ | ☐ | ☐ | ☐ | ☐ |
| 1. Professional | ☐ | ☐ | ☐ | ☐ | ☐ | ☐ | ☐ | ☐ | ☐ | ☐ | ☐ |
| 1. Relaxed | ☐ | ☐ | ☐ | ☐ | ☐ | ☐ | ☐ | ☐ | ☐ | ☐ | ☐ |
| 1. Hostile | ☐ | ☐ | ☐ | ☐ | ☐ | ☐ | ☐ | ☐ | ☐ | ☐ | ☐ |
| 1. Truthful | ☐ | ☐ | ☐ | ☐ | ☐ | ☐ | ☐ | ☐ | ☐ | ☐ | ☐ |
| 1. Honest | ☐ | ☐ | ☐ | ☐ | ☐ | ☐ | ☐ | ☐ | ☐ | ☐ | ☐ |
| 1. Arrogant | ☐ | ☐ | ☐ | ☐ | ☐ | ☐ | ☐ | ☐ | ☐ | ☐ | ☐ |
| 1. Approachable | ☐ | ☐ | ☐ | ☐ | ☐ | ☐ | ☐ | ☐ | ☐ | ☐ | ☐ |
| 1. Responsible | ☐ | ☐ | ☐ | ☐ | ☐ | ☐ | ☐ | ☐ | ☐ | ☐ | ☐ |
| 1. Logical | ☐ | ☐ | ☐ | ☐ | ☐ | ☐ | ☐ | ☐ | ☐ | ☐ | ☐ |
| 1. Spiteful | ☐ | ☐ | ☐ | ☐ | ☐ | ☐ | ☐ | ☐ | ☐ | ☐ | ☐ |
| 1. Detached | ☐ | ☐ | ☐ | ☐ | ☐ | ☐ | ☐ | ☐ | ☐ | ☐ | ☐ |
| 1. Abusive | ☐ | ☐ | ☐ | ☐ | ☐ | ☐ | ☐ | ☐ | ☐ | ☐ | ☐ |
| 1. Independent | ☐ | ☐ | ☐ | ☐ | ☐ | ☐ | ☐ | ☐ | ☐ | ☐ | ☐ |
| 1. Cruel | ☐ | ☐ | ☐ | ☐ | ☐ | ☐ | ☐ | ☐ | ☐ | ☐ | ☐ |
| 1. Poor | ☐ | ☐ | ☐ | ☐ | ☐ | ☐ | ☐ | ☐ | ☐ | ☐ | ☐ |
| 1. Empathetic | ☐ | ☐ | ☐ | ☐ | ☐ | ☐ | ☐ | ☐ | ☐ | ☐ | ☐ |
| 1. Compassionate | ☐ | ☐ | ☐ | ☐ | ☐ | ☐ | ☐ | ☐ | ☐ | ☐ | ☐ |
| 1. Patient | ☐ | ☐ | ☐ | ☐ | ☐ | ☐ | ☐ | ☐ | ☐ | ☐ | ☐ |
| 1. Outgoing | ☐ | ☐ | ☐ | ☐ | ☐ | ☐ | ☐ | ☐ | ☐ | ☐ | ☐ |
| 1. Young | ☐ | ☐ | ☐ | ☐ | ☐ | ☐ | ☐ | ☐ | ☐ | ☐ | ☐ |
| 1. Considerate | ☐ | ☐ | ☐ | ☐ | ☐ | ☐ | ☐ | ☐ | ☐ | ☐ | ☐ |
| 1. Kind | ☐ | ☐ | ☐ | ☐ | ☐ | ☐ | ☐ | ☐ | ☐ | ☐ | ☐ |
| 1. Aggressive | ☐ | ☐ | ☐ | ☐ | ☐ | ☐ | ☐ | ☐ | ☐ | ☐ | ☐ |
| 1. Trustworthy | ☐ | ☐ | ☐ | ☐ | ☐ | ☐ | ☐ | ☐ | ☐ | ☐ | ☐ |
| 1. Carefree | ☐ | ☐ | ☐ | ☐ | ☐ | ☐ | ☐ | ☐ | ☐ | ☐ | ☐ |
| 1. Reliable | ☐ | ☐ | ☐ | ☐ | ☐ | ☐ | ☐ | ☐ | ☐ | ☐ | ☐ |
| 1. Domineering | ☐ | ☐ | ☐ | ☐ | ☐ | ☐ | ☐ | ☐ | ☐ | ☐ | ☐ |
| 1. Naïve | ☐ | ☐ | ☐ | ☐ | ☐ | ☐ | ☐ | ☐ | ☐ | ☐ | ☐ |
| 1. Knowledgeable | ☐ | ☐ | ☐ | ☐ | ☐ | ☐ | ☐ | ☐ | ☐ | ☐ | ☐ |
| 1. Dithering | ☐ | ☐ | ☐ | ☐ | ☐ | ☐ | ☐ | ☐ | ☐ | ☐ | ☐ |
| 1. Confused thinker | ☐ | ☐ | ☐ | ☐ | ☐ | ☐ | ☐ | ☐ | ☐ | ☐ | ☐ |
| 1. Sporty | ☐ | ☐ | ☐ | ☐ | ☐ | ☐ | ☐ | ☐ | ☐ | ☐ | ☐ |
| 1. Warm | ☐ | ☐ | ☐ | ☐ | ☐ | ☐ | ☐ | ☐ | ☐ | ☐ | ☐ |
| 1. Emotionally unstable | ☐ | ☐ | ☐ | ☐ | ☐ | ☐ | ☐ | ☐ | ☐ | ☐ | ☐ |
| 1. Good-natured | ☐ | ☐ | ☐ | ☐ | ☐ | ☐ | ☐ | ☐ | ☐ | ☐ | ☐ |
| 1. Liar | ☐ | ☐ | ☐ | ☐ | ☐ | ☐ | ☐ | ☐ | ☐ | ☐ | ☐ |
| 1. Understanding | ☐ | ☐ | ☐ | ☐ | ☐ | ☐ | ☐ | ☐ | ☐ | ☐ | ☐ |
| 1. Lazy | ☐ | ☐ | ☐ | ☐ | ☐ | ☐ | ☐ | ☐ | ☐ | ☐ | ☐ |
|  | 0% | 10% | 20% | 30% | 40% | 50% | 60% | 70% | 80% | 90% | 100% |

1. Please **tick** to indicate the percentage of **STUDENTS** who you think possess each characteristic

|  | 0% | 10% | 20% | 30% | 40% | 50% | 60% | 70% | 80% | 90% | 100% |
| --- | --- | --- | --- | --- | --- | --- | --- | --- | --- | --- | --- |
| 1. Fun | ☐ | ☐ | ☐ | ☐ | ☐ | ☐ | ☐ | ☐ | ☐ | ☐ | ☐ |
| 1. Loud | ☐ | ☐ | ☐ | ☐ | ☐ | ☐ | ☐ | ☐ | ☐ | ☐ | ☐ |
| 1. Calm | ☐ | ☐ | ☐ | ☐ | ☐ | ☐ | ☐ | ☐ | ☐ | ☐ | ☐ |
| 1. Happy | ☐ | ☐ | ☐ | ☐ | ☐ | ☐ | ☐ | ☐ | ☐ | ☐ | ☐ |
| 1. Committed | ☐ | ☐ | ☐ | ☐ | ☐ | ☐ | ☐ | ☐ | ☐ | ☐ | ☐ |
| 1. Honourable | ☐ | ☐ | ☐ | ☐ | ☐ | ☐ | ☐ | ☐ | ☐ | ☐ | ☐ |
| 1. Drinker | ☐ | ☐ | ☐ | ☐ | ☐ | ☐ | ☐ | ☐ | ☐ | ☐ | ☐ |
| 1. Wealthy | ☐ | ☐ | ☐ | ☐ | ☐ | ☐ | ☐ | ☐ | ☐ | ☐ | ☐ |
| 1. Loyal | ☐ | ☐ | ☐ | ☐ | ☐ | ☐ | ☐ | ☐ | ☐ | ☐ | ☐ |
| 1. Professional | ☐ | ☐ | ☐ | ☐ | ☐ | ☐ | ☐ | ☐ | ☐ | ☐ | ☐ |
| 1. Relaxed | ☐ | ☐ | ☐ | ☐ | ☐ | ☐ | ☐ | ☐ | ☐ | ☐ | ☐ |
| 1. Hostile | ☐ | ☐ | ☐ | ☐ | ☐ | ☐ | ☐ | ☐ | ☐ | ☐ | ☐ |
| 1. Truthful | ☐ | ☐ | ☐ | ☐ | ☐ | ☐ | ☐ | ☐ | ☐ | ☐ | ☐ |
| 1. Honest | ☐ | ☐ | ☐ | ☐ | ☐ | ☐ | ☐ | ☐ | ☐ | ☐ | ☐ |
| 1. Arrogant | ☐ | ☐ | ☐ | ☐ | ☐ | ☐ | ☐ | ☐ | ☐ | ☐ | ☐ |
| 1. Approachable | ☐ | ☐ | ☐ | ☐ | ☐ | ☐ | ☐ | ☐ | ☐ | ☐ | ☐ |
| 1. Responsible | ☐ | ☐ | ☐ | ☐ | ☐ | ☐ | ☐ | ☐ | ☐ | ☐ | ☐ |
| 1. Logical | ☐ | ☐ | ☐ | ☐ | ☐ | ☐ | ☐ | ☐ | ☐ | ☐ | ☐ |
| 1. Spiteful | ☐ | ☐ | ☐ | ☐ | ☐ | ☐ | ☐ | ☐ | ☐ | ☐ | ☐ |
| 1. Detached | ☐ | ☐ | ☐ | ☐ | ☐ | ☐ | ☐ | ☐ | ☐ | ☐ | ☐ |
| 1. Abusive | ☐ | ☐ | ☐ | ☐ | ☐ | ☐ | ☐ | ☐ | ☐ | ☐ | ☐ |
| 1. Independent | ☐ | ☐ | ☐ | ☐ | ☐ | ☐ | ☐ | ☐ | ☐ | ☐ | ☐ |
| 1. Cruel | ☐ | ☐ | ☐ | ☐ | ☐ | ☐ | ☐ | ☐ | ☐ | ☐ | ☐ |
| 1. Poor | ☐ | ☐ | ☐ | ☐ | ☐ | ☐ | ☐ | ☐ | ☐ | ☐ | ☐ |
| 1. Empathetic | ☐ | ☐ | ☐ | ☐ | ☐ | ☐ | ☐ | ☐ | ☐ | ☐ | ☐ |
| 1. Compassionate | ☐ | ☐ | ☐ | ☐ | ☐ | ☐ | ☐ | ☐ | ☐ | ☐ | ☐ |
| 1. Patient | ☐ | ☐ | ☐ | ☐ | ☐ | ☐ | ☐ | ☐ | ☐ | ☐ | ☐ |
| 1. Outgoing | ☐ | ☐ | ☐ | ☐ | ☐ | ☐ | ☐ | ☐ | ☐ | ☐ | ☐ |
| 1. Young | ☐ | ☐ | ☐ | ☐ | ☐ | ☐ | ☐ | ☐ | ☐ | ☐ | ☐ |
| 1. Considerate | ☐ | ☐ | ☐ | ☐ | ☐ | ☐ | ☐ | ☐ | ☐ | ☐ | ☐ |
| 1. Kind | ☐ | ☐ | ☐ | ☐ | ☐ | ☐ | ☐ | ☐ | ☐ | ☐ | ☐ |
| 1. Aggressive | ☐ | ☐ | ☐ | ☐ | ☐ | ☐ | ☐ | ☐ | ☐ | ☐ | ☐ |
| 1. Trustworthy | ☐ | ☐ | ☐ | ☐ | ☐ | ☐ | ☐ | ☐ | ☐ | ☐ | ☐ |
| 1. Carefree | ☐ | ☐ | ☐ | ☐ | ☐ | ☐ | ☐ | ☐ | ☐ | ☐ | ☐ |
| 1. Reliable | ☐ | ☐ | ☐ | ☐ | ☐ | ☐ | ☐ | ☐ | ☐ | ☐ | ☐ |
| 1. Domineering | ☐ | ☐ | ☐ | ☐ | ☐ | ☐ | ☐ | ☐ | ☐ | ☐ | ☐ |
| 1. Naive | ☐ | ☐ | ☐ | ☐ | ☐ | ☐ | ☐ | ☐ | ☐ | ☐ | ☐ |
| 1. Knowledgeable | ☐ | ☐ | ☐ | ☐ | ☐ | ☐ | ☐ | ☐ | ☐ | ☐ | ☐ |
| 1. Dithering | ☐ | ☐ | ☐ | ☐ | ☐ | ☐ | ☐ | ☐ | ☐ | ☐ | ☐ |
| 1. Confused thinker | ☐ | ☐ | ☐ | ☐ | ☐ | ☐ | ☐ | ☐ | ☐ | ☐ | ☐ |
| 1. Sporty | ☐ | ☐ | ☐ | ☐ | ☐ | ☐ | ☐ | ☐ | ☐ | ☐ | ☐ |
| 1. Warm | ☐ | ☐ | ☐ | ☐ | ☐ | ☐ | ☐ | ☐ | ☐ | ☐ | ☐ |
| 1. Emotionally unstable | ☐ | ☐ | ☐ | ☐ | ☐ | ☐ | ☐ | ☐ | ☐ | ☐ | ☐ |
| 1. Good-natured | ☐ | ☐ | ☐ | ☐ | ☐ | ☐ | ☐ | ☐ | ☐ | ☐ | ☐ |
| 1. Liar | ☐ | ☐ | ☐ | ☐ | ☐ | ☐ | ☐ | ☐ | ☐ | ☐ | ☐ |
| 1. Understanding | ☐ | ☐ | ☐ | ☐ | ☐ | ☐ | ☐ | ☐ | ☐ | ☐ | ☐ |
| 1. Lazy | ☐ | ☐ | ☐ | ☐ | ☐ | ☐ | ☐ | ☐ | ☐ | ☐ | ☐ |
|  | 0% | 10% | 20% | 30% | 40% | 50% | 60% | 70% | 80% | 90% | 100% |

1. Please **circle** a number to indicate how much you agree with each of these statements

|  | Strongly Disagree Disagree Neutral Agree Agree Strongly  Disagree Somewhat Somewhat Agree |
| --- | --- |
| 1. Being a doctor is an important part of my self image | 1 2 3 4 5 6 7 |
| 1. Being a doctor is unimportant to my sense of what kind of person I am | 1 2 3 4 5 6 7 |
| 1. Being a doctor is an important reflection of who I am | 1 2 3 4 5 6 7 |
| 1. Being a doctor has very little to do with how I feel about myself | 1 2 3 4 5 6 7 |

1. Considering the group ‘**DOCTORS’**, please **circle** a number to indicate how much you agree with each of these statements

|  | Strongly Disagree Disagree Neutral Agree Agree Strongly  Disagree Somewhat Somewhat Agree |
| --- | --- |
| 1. I am a person who considers the group important | 1 2 3 4 5 6 7 |
| 1. I am a person who identifies with the group | 1 2 3 4 5 6 7 |
| 1. I am a person who feels strong ties with the group | 1 2 3 4 5 6 7 |
| 1. I am a person who is glad to belong to the group | 1 2 3 4 5 6 7 |
| 1. I am a person who sees myself as belonging to the group | 1 2 3 4 5 6 7 |
| 1. I am a person who makes excuses for belonging to the group | 1 2 3 4 5 6 7 |
| 1. I am a person who tries to hide belonging to the group | 1 2 3 4 5 6 7 |
| 1. I am a person who feels held back by the group | 1 2 3 4 5 6 7 |
| 1. I am a person who is annoyed to say I'm a member of the group | 1 2 3 4 5 6 7 |
| 1. I am a person who criticizes the group | 1 2 3 4 5 6 7 |

1. Please **circle** a number to indicate how much you agree with each of these statements

|  | Strongly Disagree Disagree Neutral Agree Agree Strongly  Disagree Somewhat Somewhat Agree |
| --- | --- |
| 1. Being a student is an important part of my self image | 1 2 3 4 5 6 7 |
| 1. Being a student is unimportant to my sense of what kind of person I am | 1 2 3 4 5 6 7 |
| 1. Being a student is an important reflection of who I am | 1 2 3 4 5 6 7 |
| 1. Being a student has very little to do with how I feel about myself | 1 2 3 4 5 6 7 |

1. Considering the group ‘**STUDENTS’**, please **circle** a number to indicate how much you agree with each of these statements

|  | Strongly Disagree Disagree Neutral Agree Agree Strongly  Disagree Somewhat Somewhat Agree |
| --- | --- |
| 1. I am a person who considers the group important | 1 2 3 4 5 6 7 |
| 1. I am a person who identifies with the group | 1 2 3 4 5 6 7 |
| 1. I am a person who feels strong ties with the group | 1 2 3 4 5 6 7 |
| 1. I am a person who is glad to belong to the group | 1 2 3 4 5 6 7 |
| 1. I am a person who sees myself as belonging to the group | 1 2 3 4 5 6 7 |
| 1. I am a person who makes excuses for belonging to the group | 1 2 3 4 5 6 7 |
| 1. I am a person who tries to hide belonging to the group | 1 2 3 4 5 6 7 |
| 1. I am a person who feels held back by the group | 1 2 3 4 5 6 7 |
| 1. I am a person who is annoyed to say I'm a member of the group | 1 2 3 4 5 6 7 |
| 1. I am a person who criticizes the group | 1 2 3 4 5 6 7 |

**Demographic information**

**(This information but it will allow us to compare the responses of different groups of people)**

1. Are you: ☐ Female

☐ Male

1. Please state your age __________________
2. Please state your year of study __________________
3. Please state your nationality __________________
4. Please state your ethnic background __________________
5. Please indicate the specialty career path you intend to pursue as a doctor:

☐ I haven’t decided yet

☐ Medicine

☐ Surgery

☐ Obstetrics and Gynaecology

☐ Paediatrics

☐ Psychiatry

☐ General Practice

☐ Laboratory medicine

☐ Other (please state) __________________

1. Please describe briefly below an example of a time where you felt **most like** a doctor

|  |
| --- |

1. We may contact you in the future to complete a follow-up questionnaire, and would like to be able to match your responses to any future responses. However, we would also like to be sure that your data remains anonymous. To enable this we would be grateful if you could answer the following questions.
2. What is the INITIAL of your first name? __________
3. What is the DAY of your birthday? (e.g., 22nd) __________
4. What are the FIRST TWO LETTERS of the town / city where you were born? __________

***Thank you for taking part.***

*Please make sure the consent form is detached from the questionnaire before returning both it and this questionnaire.*

*If you have any questions about the study please email [lead researcher email]*
